# Cloning, expression and purification of recombinant dermatopontin in *Escherichia coli*

**DOI:** 10.1371/journal.pone.0242798

**Published:** 2020-11-30

**Authors:** Trikkur Madom Seetaraman Amritha, Shubham Mahajan, Kumar Subramaniam, Yamini Chandramohan, Anuradha Dhanasekaran

**Affiliations:** 1 Centre for Biotechnology, Anna University, Chennai, Tamil Nadu, India; 2 SRM Institute of Science and Technology, Chennai, Tamil Nadu, India; USDA-ARS Southeast Area, UNITED STATES

## Abstract

Dermatopontin (DPT) is an extracellular matrix (ECM) protein with diversified pharmaceutical applications. It plays important role in cell adhesion/migration, angiogenesis and ECM maintenance. The recombinant production of this protein will enable further exploration of its multifaceted functions. In this study, DPT protein has been expressed in *Escherichia coli (E*.*coli)* aiming at cost effective recombinant production. The *E*.*coli* GJ1158 expression system was transformed with constructed recombinant vector (pRSETA-DPT) and protein was expressed as inclusion bodies on induction with NaCl. The inclusion bodies were solubilised in urea and renaturation of protein was done by on-column refolding procedure in Nickel activated Sepharose column. The refolded Histidine-tagged DPT protein was purified and eluted from column using imidazole and its purity was confirmed by analytical techniques. The biological activity of the protein was confirmed by collagen fibril assay, wound healing assay and Chorioallantoic Membrane (CAM) angiogenesis assay on comparison with standard DPT. The purified DPT was found to enhance the collagen fibrillogenesis process and improved the migration of human endothelial cells. About 73% enhanced wound closure was observed in purified DPT treated endothelial cells as compared to control. The purified DPT also could induce neovascularisation in the CAM model. At this stage, scaling up the production process for DPT with appropriate purity and reproducibility will have a promising commercial edge.

## Introduction

Dermatopontin (DPT) is an extracellular non collagenous protein with its expression conserved in mammals and invertebrates. It is expressed in various human tissues like skeletal muscle, bone, cartilage and skin [1]. This protein was initially isolated from bovine dermal extract as it got co-eluted during the purification of decorin protein [2]. Mammalian dermatopontin have five loop structures composed of five intramolecular disulphide bonds [3]. The wide range expression of this protein in different mammalian tissues, suggests that it has multiple functions. Apart from its major function in maintaining the functional tissue integrity of skin ECM architecture, it has also found to have major role in promoting cell adhesion, wound healing, osteogenic differentiation of adipose derived stem cells, and angiogenesis [4–7]. It is also known to interact with Transforming growth factor beta 1 (TGFβ1) and Decorin proteins to modulate various processes [8]. DPT is also a major player in collagen fibrillogenesis process and is found distributed on the surface of collagen fibres [1]. This multifunctional protein exhibits potential for many applications particularly in wound healing, tissue engineering and as an effective cosmetic formulant. Although, the ideal way of obtaining the protein is from its original source such as mammalian tissues like skin, or from shells of invertebrate, it is not suitable because of various constraints like availability, time and labor. Therefore, various heterologous protein production systems are preferred to produce the protein in *in-vitro* conditions. Previously c-DNA of DPT gene has been cloned to goldfish *Carassius auratus* [9]. However, the ease of use, lesser doubling time and scalability makes prokaryotic bacterial systems to be more advantageous to be used for heterologous protein expression. One of the most used host as model organism for eukaryotic protein expression is *Escherichia coli* [10]. In this study, we have cloned and expressed DPT gene in a salt inducible promoter containing *E*.*coli* GJ1158 expression system using pRSETA vector aiming cost effective production and it has not been reported earlier. Cloning in pRSETA allows protein to be expressed with a N-terminal histag and allows protein purification from crude lysate to be achieved using affinity column chromatography [11]. The biological activity of the protein to support collagen fibril formation and *in vitro* wound healing was analyzed and the purified DPT showed functional activity comparable to standard DPT.

## Materials and methods

### Chemicals, reagents, strains, plasmid, and cell line

Restriction enzymes BamH1, HindIII, reaction buffer, PCR components: 5X Master mix, Rapid Ligation kit and Phusion polymerase were purchased from New England Biolabs, USA. Primers were obtained from Eurofins, Banglore, India. Plasmid isolation kit, PCR Clean up kit, and Gel Extracton kit were obtained from Qiagen, Hilden, Germany. Immobilized metal affinity chromatography (IMAC) Sepharose column was purchased from GE Healthcare Amersham, U.K. Amicon Centrifugal Filters were procured from Merck, India. Nitrocellulose membrane and Substrates—H_2_O_2_ and luminol were purchased from Bio-Rad India. DNA ladder and protein ladder was procured from Jena Bioscience, Germany. DPT standard was purchased from R&D Biosystems, USA. DPT mouse polyclonal antibody (SC-376863) was procured from Santacruz biotechnology, USA. The chemicals used in media preparation, buffer and reagents were of analytical grade and purchased from Himedia, Mumbai, India. DPT synthetic gene construct was obtained from Genscript, USA. pRSETA expression vector was obtained from Invitrogen Life Technologies. The bacterial strain *E*. *coli* DH5α was used as maintenance strain for propagation of recombinant vector and *E*. *coli* GJ1158 was used as expression host. The strain was procured from the Centre for Cellular and Molecular Biology (CCMB), India. The EA. hy926 endothelial cells were procured from ATCC(USA). The cells were cultured in 25 cm^2^ tissue culture flask in Dulbecco’s Modified Eagles medium containing 10% Fetal Bovine Serum FBS (Invitrogen) and 1% antibiotic Penicillin-Streptomycin (100 units/ml) and maintained in 5% CO_2_ incubator at 37°C.

### Ethics approval

The recombinant study was conducted in accordance with the proper guidelines and regulations approved by the Biosafety Institutional Ethical Committee of Anna University (BT/17/008/94–PID).

### Recombinant vector construction

The human Dermatopontin gene sequence was obtained from NCBI (National Center for Biotechnology Information) search engine (Accession No. NM_001937.5) and was submitted for synthetic gene construction.

The DPT gene was amplified from the synthetic construct by reverse transcriptase PCR (2720 Thermal cycler Applied Biosystems, USA) using Phusion polymerase enzyme. Amplification was done using gene specific primers, h-DPT forward: ^5^’*CGCGGATCC***ATG**CAGTATGGCGATTATG^3^’ and h-DPT reverse primer ^5’^*CCCAAGCTT*CTAAACATTTGCAAATTCACAGTCGTATTC^3^’. The forward primer contained Bam HI site with its flanking sequence (given in italics) and ATG site (given in bold) incorporated for amplification of coding region. The reverse primer had the restriction site Hind III incorporated with its flanking sequence (given in italics). The PCR reaction was carried out under the following optimised conditions: initial denaturation at 95°C for 4 min, followed by 30 cycles of denaturation at 95°C for 30 s, annealing at 62°C for 30 s, and elongation at 72°C for 30 s. The amplified product was electrophoresed on 1% agarose and gel purified. The DPT gene and the pRSETA vector were digested with the enzymes Bam H1 and Hind III and ligated. The recombinant vector construct which has DPT gene incorporated in proximity to His tag sequence in pRSETA vector was transformed into *E*.*coli* DH5α competent cells and grown in Luria Bertani medium (LB) containing ampicillin (100 μg/ml). The transformants bearing the recombinant vector were confirmed by colony PCR, restriction digestion analysis and by sequencing of recombinant vector. The theoretical molecular weight of the His-tagged DPT protein was analyzed using the software (https://web.expasy.org/compute_pi/) and was predicted to be 26 kDa.

### Expression of recombinant DPT

The *Escherichia coli* strain GJ1158 was transformed with recombinant vector (pRSETA-DPT) using standard procedures [12] and spread plated on Ampicillin (100 μg/ml) containing LB-ON (LB media without NaCl) agar plates. Positive transformants were confirmed by colony PCR. A single colony from the transformed *E*.*coli* was picked and inoculated in 100 ml Luria Bertani medium without sodium chloride (LB-ON) (Tryptone 1% (w/v), Yeast extract 0.5% (w/v)) containing Ampicillin antibiotic. The culture was grown overnight at 180 rpm, 37°C in an orbital shaker. About 1% (v/v) of overnight culture was inoculated into 1000 ml of fresh LB-ON media and was grown at 37°C with shaking. On reaching 0.6 OD_600_, NaCl at a final concentration of 300 mM was added to media for protein expression induction and culture was incubated at 37°C. After 4 hours of incubation, the cells were harvested by centrifuging at 6500 g for 15 min at 4°C. The cell pellet was resuspended in lysis buffer (50 mM Tris, pH 8.0, 500 mM NaCl, 2% glycerol (v/v), 1mM β-mercaptoethanol) and homogenized (600–800 bar for 5 cycles, 4°C). The cell lysate obtained was centrifuged at 20000 g for 15 min at 4°C followed by a wash with lysis buffer. The inclusion bodies were solubilised in lysis buffer containing 8 M urea. The sample was incubated at room temperature for 2 h and centrifuged at 20000 g for 20 min at 4°C. The supernatant fraction containing the solubilized protein was collected and protein concentration was estimated by Bradford method [13]. The samples collected at each stage of expression were loaded equally (10μl) on to 12% (w/v) SDS gel to analyze the protein expression. The bands were visualized on staining with Coomassie Brilliant blue R-250. The Bio-Rad pre-stained protein marker (Precision Plus Protein™ Dual Color Standards, Bio-Rad, India) was also used to determine the molecular weight of protein.

### Purification of recombinant DPT

DPT protein was purified by Immobilized metal affinity chromatography using Nickel activated Sepharose column. The column was activated using 0.1 M nickel sulphate and pre-equilibrated with lysis buffer and 8 M urea containing lysis buffer. 20 ml of urea solubilized protein lysate of concentration 406.16 μg/ml was loaded on to column at flow rate of 0.5 ml/min and flowthrough from the column was collected. A column wash was given to remove any unbound protein. Refolding was done by gradually decreasing the urea concentration from 8 M to 0 M. Each gradient concentration (7 M to 0 M) having a final volume of 20 ml was prepared in lysis buffer and was applied to column sequentially at a constant flow rate of 1 ml/min, thereby gradually decreasing urea exposure. The final column wash was with lysis buffer containing 20 mM imidazole. The renatured protein was eluted at 150 mM imidazole. The various elute fraction of recombinant DPT (rDPT) was pooled and dialyzed against buffer A (50 mM Tris, pH 8.0, 500 mM NaCl, 2% glycerol (v/v)) overnight at 4°C for gradual removal of β-mercaptoethanol and sample was concentrated by Amicon centricon centrifugal filter (3kDa cut off membrane) to remove any residual imidazole. The fractions were confirmed for protein expression under reducing conditions using 12% SDS gel (w/v).

### Western blotting

The purified rDPT protein (26.8 μg) and standard DPT (800 ng) were separated using 12% SDS-PAGE (w/v) and electroblotted onto nitrocellulose membrane using a trans blot apparatus (Bio-Rad, CA, USA). The membrane was blocked with 5% skimmed milk (w/v) for 1 h and was incubated with diluted mouse polyclonal DPT antibody (1:1000) overnight at 4°C. After washing the membrane with 1X TBST, it was incubated with 1:5000 dilution of horseradish peroxidase (HRP) conjugated secondary antibody at room temperature for 1 h. The membrane was washed and the bands developed on addition of chemiluminescence reagent was visualised in Odyssey Fc imager LI-COR, USA.

### Collagen fibril assay

The activity of purified rDPT to accelerate collagen fibril formation was evaluated by the collagen fibrillogenesis assay. The assay was performed according to procedure given by MacBeath et al. [14] with slight modifications. To perform the assay, 500 μg/ml of rat tail collagen (Sigma, USA) and the Fibril formation buffer (60 mM Na_2_HPO_4_, 60 mM TES (N- [Tris (hydroxymethyl) methyl]-2-aminoethanesulfonic acid), 270 mM NaCl (w/v), pH 7.4) were added in equal volume ratio (1:1) to the control as well as test wells in 96 well ELISA plate. The ELISA plate reader (BioTek Synergy HT, USA) was equilibrated to a set temperature of 34°C. All the components were also preincubated separately at 34°C. The purified rDPT and standard DPT, both diluted separately with fibril buffer to 25 μg/ml concentration was added to test wells such that volume of fibril buffer was maintained same in all wells. The buffer A was added to control wells and water was added to maintain a final volume of 200 μl in all wells. The rate of fibril formation was monitored based on turbidity measurement at 313 nm in ELISA microplate reader at one-minute time interval for 80 minutes. The experimental graph was characterized based on total turbidity change and lag phase duration.

### Wound healing assay

The wound healing activity of purified rDPT was evaluated using EA. hy926 endothelial cells. The assay was performed in 6 well plates seeded with equal density of 2*10^5^ cells /well. On attaining confluency, a scratch wound was made using 200 μl pipette tip and the cells were washed with phosphate buffered saline (PBS). It was replaced with fresh serum free media containing the different treatments. The cells were treated with fresh serum free media containing purified rDPT (20 ng/ml). Standard DPT (20 ng/ml) and Vascular endothelial growth factor (VEGF– 20 ng/ml) were used as positive controls and the cells treated with buffer A was taken as control. The migration of cells post treatment were captured at 0, 8 and 24 h using phase contrast microscope (Nikon Eclipse TS 100). The area of wound closure was measured using ImageJ software at different time periods and graph was plotted. The percentage of wound closure was calculated as given by Krishnaswamy et al [6]:

% wound closure = [(Initial wound area—Final wound area) / Initial wound area] * 100.

### Chick Chorioallantoic Membrane (CAM) angiogenesis assay

Fertilized white leghorn chicken eggs (4 to 5 days old) were purchased from Government poultry station, Potheri, Chennai, India. The eggs were carefully cut open and transferred in sterile petri dishes. Sterile papers discs soaked in purified rDPT (20 ng/ml), standard DPT (20 ng/ml) and VEGF (20 ng/ml) were placed on the developing CAM (chorioallantoic membrane) and incubated at 37°C in a humidified atmosphere. Paper discs soaked in buffer A was used as control and VEGF has been used as a positive control. Photographs of the treated CAM was taken at 0 h and 8 h of incubation using Nikon Coolpix camera (Olympus India Pvt Ltd, New Delhi, India) adapted to a stereomicroscope.

### Data analysis

All experiments were performed in triplicates and data were expressed as mean ± standard deviation. Significance between groups was analyzed by One-way analysis of variance (ANOVA) using GraphPad Prism (version 8.4.3 GraphPad Software, San Diego, USA). p<0.05 was considered statistically significant.

## Results

### Construction of recombinant vector

**Fig 1:**
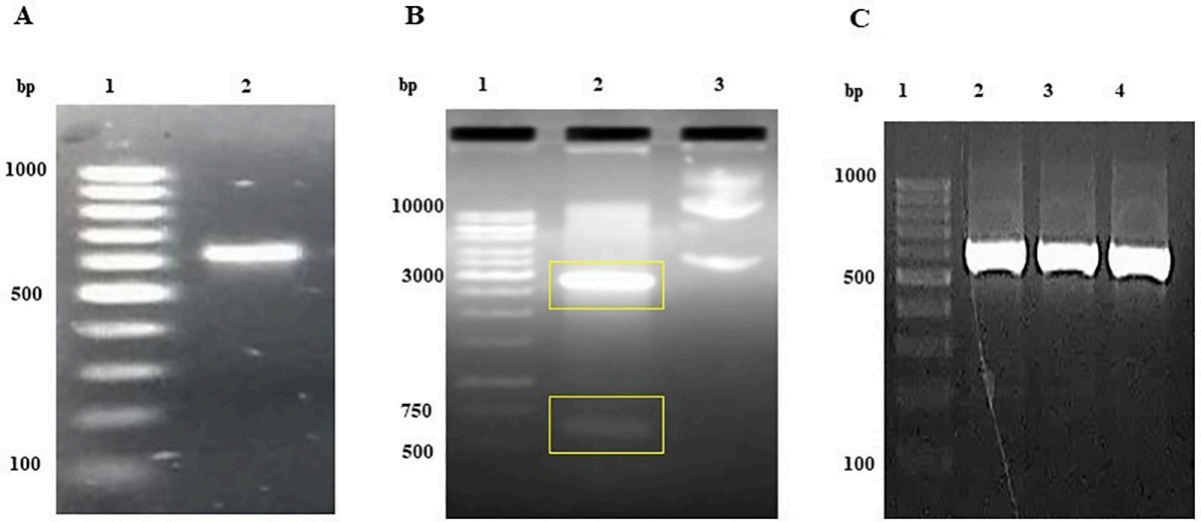
PCR amplification of Dermatopontin gene and transformation confirmation by double digestion of recombinant vector and colony PCR. (A) Lane 1: 100 bp DNA ladder; Lane 2: Dermatopontin gene fragment of 614 bp size amplified from the synthetic construct using gene specific primers (B) Lane 1: 1kb DNA ladder; Lane 2: Restriction digestion of recombinant vector (pRSETA- DPT) with enzymes Bam HI and Hind III resulted in a vector fragment and DPT gene fragment; Lane 3: uncut recombinant vector (C) Lane 1: 100 bp DNA ladder; Lane 2–4: Dermatopontin gene amplified from transformed clone by colony PCR.

The amplification of DPT gene by PCR showed single 614 bp (**Fig 1A**) band on agarose gel. The proper insertion of gene in the pRSETA vector was confirmed by double digestion of recombinant vector with Bam HI and Hind III restriction enzymes (**Fig 1B**) and by sequencing of recombinant vector. The digestion produced two fragments on the gel, the top band corresponds to 2.9 kb pRSETA vector and bottom band corresponds to DPT insert. The Dermatopontin gene (**Fig 1C**) amplified from randomly selected transformed clones by colony PCR confirmed the successful ligation of gene with vector construct. The pRSETA vector as well as DPT gene was double digested by 0.5 μl of 1unit of BamH1 and Hind III enzyme on incubation at 37°C in 1 h for recombinant vector construction **(S1 Fig).**

### Recombinant DPT expression and purification

**Fig 2:**
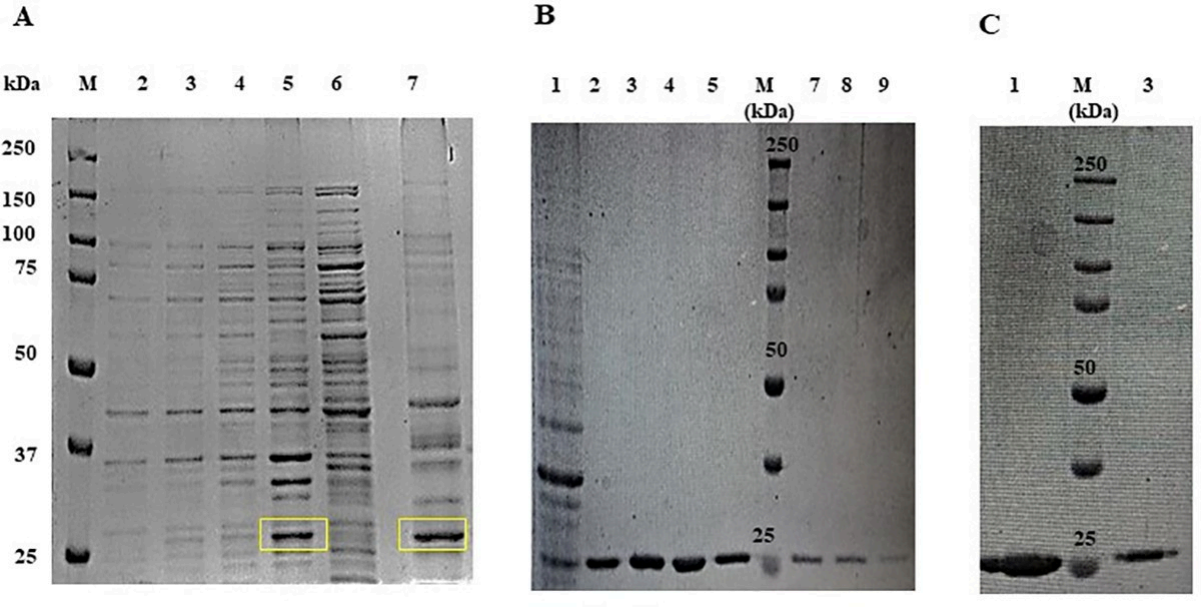
SDS PAGE analysis of recombinant Dermatopontin (rDPT) expressed in *E*.*coli* GJ1158 host as inclusion bodies and purified by affinity chromatography. (A) Lane 1: protein marker; Lane 2: protein lysate of host transformed with empty vector (pRSETA); Lane 3: induced host transformed with recombinant vector; Lane 4: soluble protein fractions obtained after homogenization of induced host cells transformed with recombinant vector; Lane 5: insoluble protein fractions obtained after homogenization of induced host cells transformed with recombinant vector; Lane 6: uninduced host transformed with recombinant vector; Lane 7: urea solubilized inclusion body fraction (B) Lane 1: Flow through from nickel activated Sepharose column; Lane 2,3,4,5,7,8,9: Purified refolded rDPT eluted at 150 mM imidazole concentration; Lane 6: protein marker (C) Lane 1 & 3: centricon concentrated rDPT elutes; Lane 2: protein marker.

The expression of DPT protein having a molecular weight of 26 kDa was observed when *E*.*coli* GJ1158 culture containing recombinant vector was induced with 300 mM NaCl (**Fig 2A**). DPT protein expression was prominent only in the insoluble inclusion body fraction (lane 5 of **Fig 2A**). There was no expression observed in uninduced *E*.*coli* GJ1158 host transformed with recombinant vector and also in induced host culture transformed with empty vector (pRSETA). The protein was expressed as fusion protein with a His-tag, along with vector regions providing cleavage sites and this contributes to additional 4 kDa to its molecular weight. This observed protein molecular weight of 26 kDa also matched the theoretical protein molecular weight predicted. DPT protein expression was prominent only in the insoluble fraction and it was solubilised using 8 M urea which helps to disrupt the covalent interactions. The urea solubilised inclusion bodies (lane 7 of **Fig 2A**) were purified in nickel activated Sepharose column. The protein bound in column was subject to refolding by gradually decreasing the urea concentration to 0 M. The renatured protein was eluted at 150 mM imidazole concentration. The percentage of refolded DPT eluted from the column was 55.7%. All the eluted fraction (**Fig 2B**) from the column showed a single sharp band in the size range of 26 kDa when run on SDS-gel. **Fig 2C** shows the DPT protein fraction obtained after centrifugal filtration. A significant final yield of 1.905 mg/g dry weight biomass of purified protein was obtained on induction of 1 litre *E*.*coli* GJ1158 host culture transformed with recombinant vector.

### Western blot analysis

**Fig 3:**
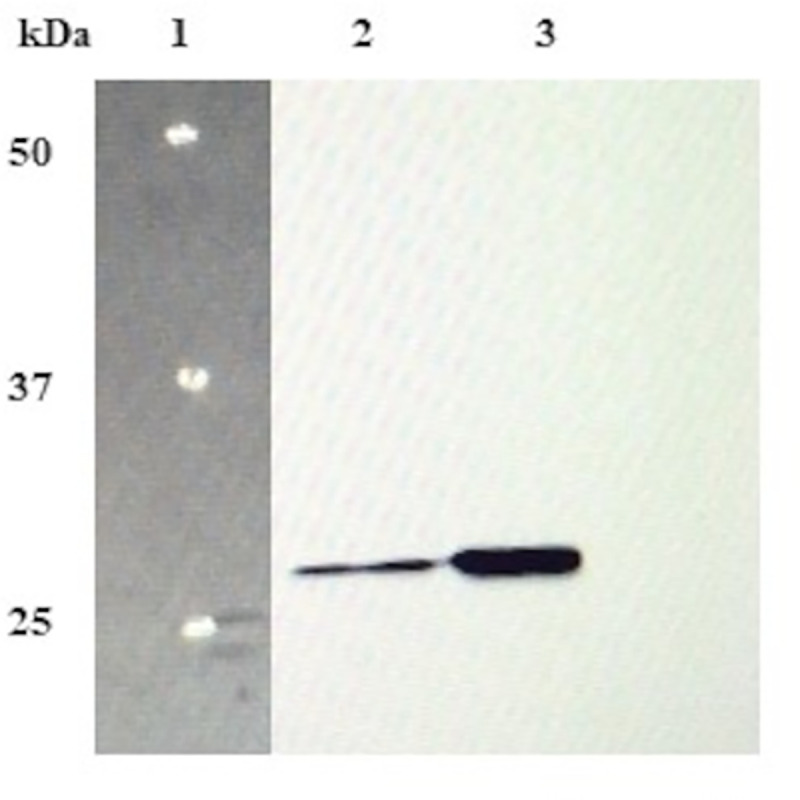
Western blot analysis of Dermatopontin. Lane 1: protein marker; Lane 2: standard DPT; Lane 3: rDPT.

The western blot analysis (**Fig 3**) done by probing with DPT specific antibody further confirmed the integrity of DPT protein as observed by a single prominent band corresponding to 26 kDa.

### Collagen fibril assay

**Fig 4:**
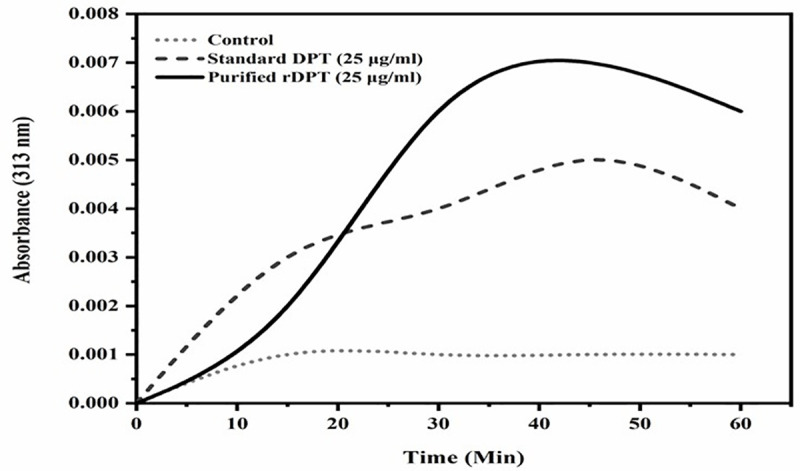
Collagen fibril assay. The graph represents the effect of Dermatopontin on collagen fibrillogenesis. Dermatopontin induces collagen fibril formation and the rate of fibril formation was significantly enhanced in DPT treated wells compared to control. Collagen I was mixed at a constant concentration of 500 μg/ml with equal concentrations (25 μg/ml) of purified recombinant Dermatopontin and standard. Equal volume of fibril formation buffer was added to all wells. Fibrillogenesis was monitored by optical density-based turbidity measurement at 313 nm.

The collagen fibril assay enables turbidimetric quantification of DPT activity. The warm start technique adopted by maintaining a constant temperature of 34°C throughout the assay helps in initiation of fibrillogenesis process immediately on addition of components. From **Fig 4**, it can be observed both rDPT and standard DPT show similar pattern of fibrillogenesis. A small lag phase was observed in control and purified rDPT treated wells. It was observed that fibrillogenesis increases with time for both control and DPT added sample. Both the purified and standard DPT treated wells showed a higher absorbance turbidity value compared to control. The fibril formation reached a saturation point around 60 min. The maximum turbidity attained by purified DPT and standard DPT was 0.05 and 0.07 respectively. The rate of fibril formation was significantly higher in DPT treated wells compared to control (p<0.05).

### Wound healing assay

**Fig 5:**
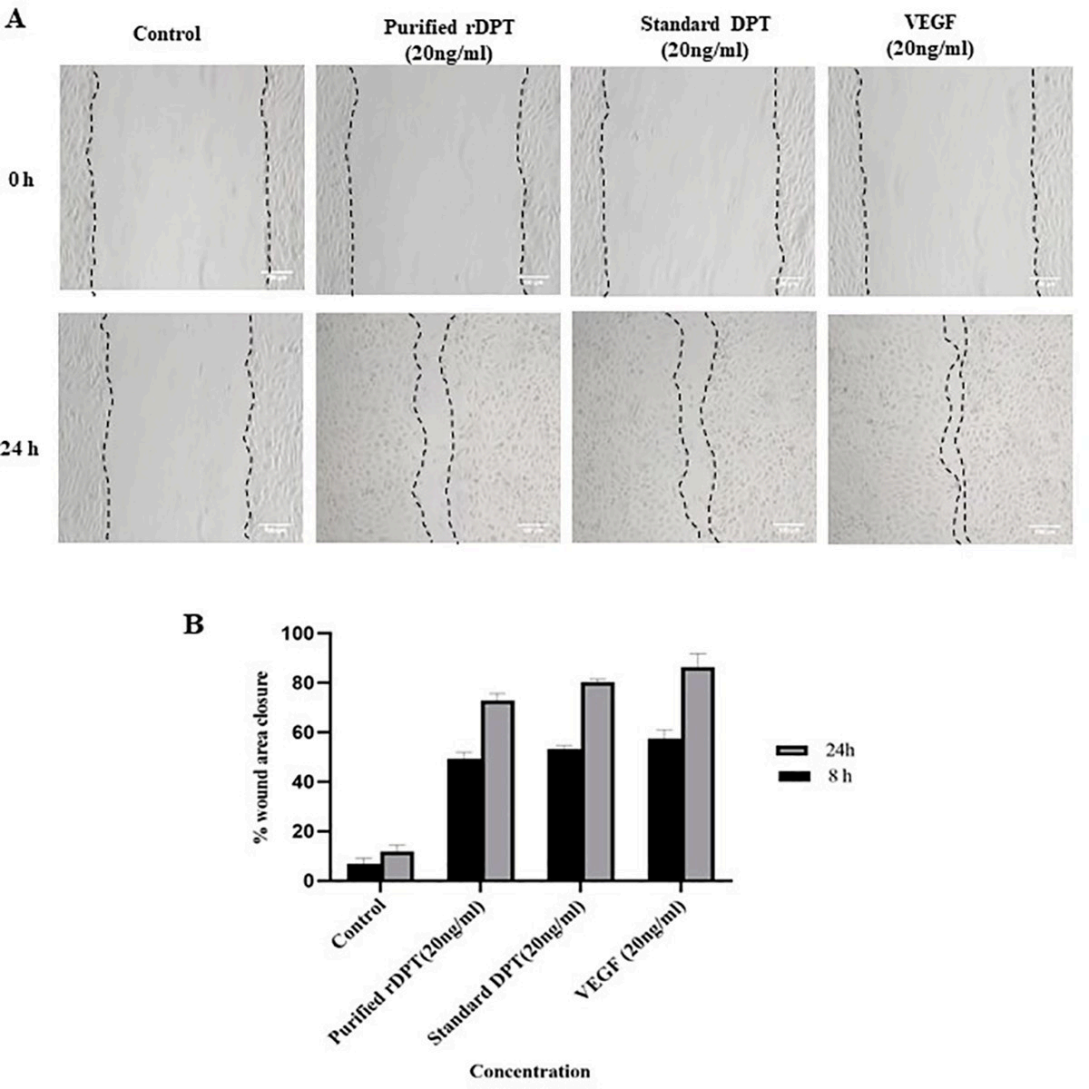
Effect of Dermatopontin (DPT) and Vascular endothelial growth factor (VEGF) on endothelial cell migration. (A) Microscopic images represent the migration pattern of endothelial cells (EA. hy926) treated with equal concentration (20 ng/ml) of purified rDPT, standard DPT and VEGF (positive control) compared to control after a scratch wound was created on confluent endothelial cells cultured on 6-well tissue culture plate. Images of the treated and control wells taken at time 0 h and 24 h post treatment are shown. Scale: 100μm. (B) Graphical representation of percentage wound closure after treatment with equal concentrations (20ng/ml) of purified rDPT, standard DPT and VEGF (positive control) compared to control. Values are expressed as mean ± standard deviation. The percentage wound closure in all treatment groups were statistically significant compared to control group (p<0.05).

This assay evaluates the wound healing efficacy of the purified rDPT based on scratch wound area covered with time. From the (**Fig 5A and 5B**), it can be observed that DPT was able to promote migration of EA. hy926 cells. At the end of 24 h, the cells treated with purified rDPT migrated to 73% wound area and those treated with standard DPT covered 80% wound area whereas in control cells only 12% area was covered. In VEGF treated cells, 86% wound closure was observed. The percentage wound closure was significantly higher for DPT treated and VEGF treated cells compared to control. When treated with rDPT, the percentage of wound closure increased and this was comparable to the results obtained with standard DPT. There were no significant differences between the standard and purified rDPT treated groups indicating that their functional activity was similar. The percentage wound closure in all the treated groups were significantly higher when compared to control group (p<0.05).

### Chick Chorioallantoic Membrane (CAM) angiogenesis assay

**Fig 6:**
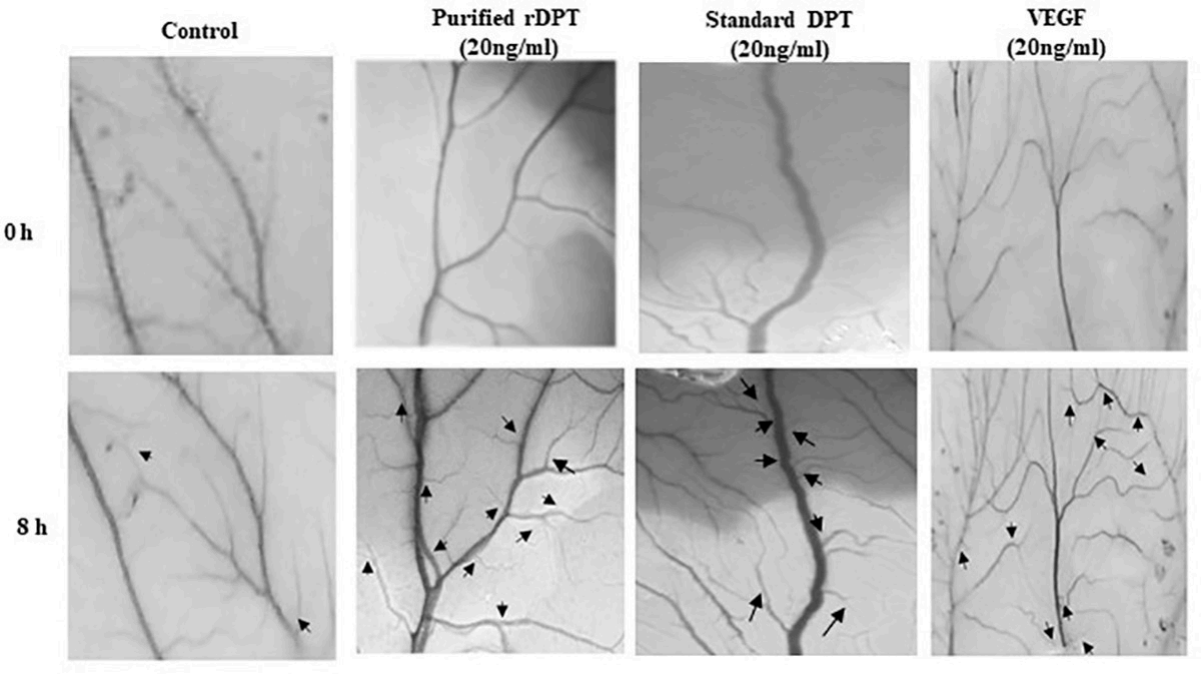
Angiogenesis assay using Chick Chorioallantoic Membrane (CAM) model. Photographic images of CAM taken at 0 h and 8 h after treatment with equal concentrations (20ng/ml) of purified recombinant Dermatopontin, standard Dermatopontin and Vascular Endothelial Growth Factor. Arrows denote the newly formed capillaries.

CAM assay was performed using developing chick embryo model and the images of treated CAM taken at 0 h and 8 h are shown in **Fig 6**. It was observed that DPT treatment, induced sprouting of small capillaries from the pre-existing vessel. In comparison to control, increased neovascularization could be observed in DPT treated CAM at the end of 8 h. The positive control, VEGF was also shown to induce neovascularization by sprouting angiogenesis. The results confirm that DPT can induce angiogenesis and this result also is in compliance with the previous report by Krishnaswamy et al. [6] that DPT augments angiogenesis and has pivotal role in angiogenesis.

## Discussion

Dermatopontin is a non-collagenous extracellular matrix protein having significant different roles in the various tissues it is expressed in humans. Recently, DPT has also been reported to be used as a therapeutic agent for metabolic disorders [15]. Recombinant production of this protein using a cost-effective strategy will be useful for its various biotechnological application and for further research focused on its function. Hence, in this study, DPT protein production was done using simple prokaryotic system of *E*.*coli* GJ1158 which have been successful in producing various eukaryotic proteins like erythropoietin and Streptokinase [16]. This strain has a chromosomally integrated T7RNA polymerase gene present downstream of an osmotically inducible proU promoter [17–18] and is known to improve folding and activity of expressed recombinant protein [19]. The use of non-toxic NaCl for induction and the low cost of this expression strain make it favourable for cost effective eukaryotic protein production. The expression vector has also been previously used for production of eukaryotic protein in *E*.*coli* strains [20]. In the pRSETA vector, the gene is placed under the T7 promoter system and it allows for protein to be expressed with a N-terminal histidine tag. The presence of His tag is advantageous as it minimizes chances for protein aggregation that can occur with intermolecular interaction when the protein is bound to column [21]. Eukaryotic protein has been expressed with His-tag before and has not reported to affect the activity or structure of protein [22–24]. Many commercially important eukaryotic proteins have been expressed and purified in prokaryotic host systems. Recently, insulin protein was expressed in the intracellular fraction of *E*.*coli* BL21 and the yield obtained was 520.92 mg/l [25]. Similarly, growth factors like Platelet-derived growth factor (PDGF) have also been expressed under T7 promoter system in *E*.*coli* BL21 (DE3), purified and made available for commercial uses [26]. In this study, we had also attempted incubating the transformed culture at lower temperatures of 16°C and 28°C after induction, as these conditions are known to favour soluble protein expression. But at all temperatures, expression was observed only in the insoluble fraction and better expression was obtained when induced culture was incubated at 37°C.

The type of chromatography adopted for protein purification depends on the nature of protein [27, 28]. In this study, affinity chromatography-based purification was performed and high purity DPT protein could be obtained. The recovery of active DPT protein was achieved by on-column refolding procedure. The gradual linear reduction of denaturant allows for protein renaturation in a gentle environment [21]. The use of additives like glycerol contributes to protein stability during the refolding process [29]. Similar on-column refolding strategy and purification has been adopted earlier for production of eukaryotic proteins like Interferon-lambda 1 and extracellular superoxide dismutase enzyme expressed in *E*.*coli* [23, 30]. To ensure that the refolded protein was functional, various biological assays were performed. The collagen fibrillogenesis assay confirmed the collagen fibril formation inducing property of Dermatopontin. These results are in agreement with the previous report that dermatopontin purified from porcine skin accelerated type I collagen fibrillogenesis in vitro [14]. The crosslinking of collagen fibres by DPT may be because of the presence of N-Y-D sequence in the protein, which is also a conserved sequence in many amine oxidase (lysyl oxidase) that helps in formation of covalent crosslinks between collagen molecules [9]. In mammals, Dermatopontin is associated with collagen fibril organization and collagen fibrillogenesis which are important processes for tissue healing to occur. It has been reported that DPT deficient mice exhibits disrupted organization of collagen fibres in skin as well as in cornea [31, 32]. The load–bearing and stress shielding effect are brought about by these collagen fibres during healing process and its improper assembly can lead to fibrosis [33]. Tissue wound contraction which requires proper assembly and arrangement of collagen fibres is an important requisite process in extracellular matrix (ECM) establishment phase of wound healing. Dermatopontin which remain associated with collagen fibres *in-vivo* thus contributes largely to ECM establishment phase as it can initiate and enhance the collagen fibril formation process.

DPT has been proved to influence migration of keratinocytes and EA. hy926 endothelial cells [5, 6]. The observed results also showed an increase in migration of cells when treated with DPT as compared to control. The migration of cells treated with both rDPT and standard were comparable. DPT is also known to enhance cell adhesion in various cell types [7, 34–36]. The angiogenic property of Dermatopontin was confirmed by CAM assay. Angiogenesis involves sprouting of new vessels from pre-existing ones and it is a requisite during various physiological process like fetal development, wound healing etc. During the wound healing process, angiogenesis causes sprouting of blood vessel capillaries in injured area where it gets organized into a vascular network over the wound tissue. The gradual reduction of blood vessel density occurs as collagen starts accumulating in the tissue to be healed [37]. The Vascular endothelial growth factor (VEGF) is an angiogenic factor that triggers the formation of new blood vessels. It has been reported that the angiogenesis enhancing activity of DPT might be by its ability to induce migration of endothelial cells and also other functions that are essential for development of vascular network in vivo [6]. All these characteristic properties of DPT make it appropriate for its tissue engineering and wound healing applications. Mammalian dermatopontin have conserved R-G-A-T integrin binding sequence and recognition of these sites by various integrin receptors expressed by endothelial may also contribute to activity. The presence of histidine tag did not seem to hinder the activity of purified protein. Although protein was not obtained in soluble fraction, it was effectively refolded and showed activity comparable to that of standard DPT. The present study gives a promising foundation for recombinant production of DPT in prokaryotic system for its utilisation in various applications like bone tissue engineering [4], wound healing particularly for cutaneous tissue regeneration [5, 6] and also as an effective cosmetic anti-aging formulant [38]. It has been proved to be effective as therapeutic for metabolic disorders [15] and also has been reported to be vital for ventricular remodelling after myocardial infarctions [35]. The yield and cost effectiveness could be further improved by engineering the strain or the vector to obtain soluble protein expression.

## Conclusion

In this study, an attempt has been made to produce recombinant Dermatopontin (DPT) protein from prokaryotic *E*.*coli* GJ1158 expression system for its various therapeutic applications. Results of this study show that human DPT protein can be produced from simple prokaryotic system like *E*.*coli* with this recombinant production strategy. The yield obtained was satisfactory and the purified protein showed comparable biological activity to that of standard DPT and further preclinical studies will prove the efficacy of the therapeutic protein. Production of recombinant DPT protein from a feasible source will be an added advantage than its procurement from its origin tissue samples like skin as it is a more tedious process. The scale up of protein for large scale production applying various bioprocess strategies for overproduction, can also be considered to provide a commercially feasible niche owing to the promising activity results obtained in lab scale. As a future direction, we are working towards strategies to obtain soluble expression of protein and scale up.

## Supporting information

S1 Fig Double digestion of expression vector and Dermatopontin gene under optimized conditions. (**A)** Lane 1: 1kb DNA ladder; Lane 2: uncut pRSETA vector; Lane 3 &4: pRSETA vector digested with 0.5 μl of Bam HI and Hind III enzyme at 37°C for 0.5 h and 1 h respectively, **(B)** Lane 1: 100 bp DNA ladder; Lane 2: uncut Dermatopontin gene; Lane 3: empty lane; Lane 4–6: Dermatopontin gene digested with 0.5 μl of Bam HI and Hind III enzyme at 37°C for 1 h.
(TIF)

S1 Raw images Image 1A corresponds to Fig 1A in manuscript. PCR amplification of Dermatopontin gene. Lane 1: 100 bp DNA ladder; Lane 2: Dermatopontin gene fragment of 614 bp size amplified from the synthetic construct using gene specific primers. Image 1B corresponds to Fig 1B in manuscript. Transformation confirmation by double digestion of recombinant vector. Lane 1: 1kb DNA ladder; Lane 2: Restriction digestion of recombinant vector (pRSETA- DPT) with enzymes Bam HI and Hind III resulted in vector fragment and DPT gene fragment; Lane 3: uncut recombinant vector. Image 1C corresponds to Fig 1C in manuscript. Transformation confirmation by colony PCR. Lane 1: 100 bp DNA ladder; Lane 2–4: Dermatopontin gene amplified from transformed clone by colony PCR. Image 2A corresponds to Fig 2A in manuscript. SDS PAGE analysis of rDPT expressed in *E*.*coli* GJ1158 host as inclusion bodies. Lane 1: protein marker; Lane 2: Protein lysate of host transformed with empty vector (pRSETA); Lane 3: induced host transformed with recombinant vector; Lane 4: soluble protein fractions obtained after homogenization of induced host cells transformed with recombinant vector; Lane 5: insoluble protein fractions obtained after homogenization of induced host cells transformed with recombinant vector; Lane 6: uninduced host transformed with recombinant vector; Lane 7: urea solubilised inclusion body fraction. Image 2B corresponds to Fig 2B in manuscript. SDS PAGE analysis of rDPT purified by affinity chromatography. Lane 1: Flow through from nickel activated Sepharose column; Lane 2,3,4,5,7,8,9: Purified refolded rDPT eluted at 150 mM imidazole concentration; Lane 6: protein marker. Image 2C corresponds to Fig 2C in manuscript. SDS PAGE analysis of centricon concentrated DPT protein. Lane 1 & 3: centricon concentrated rDPT elutes; Lane 2: protein marker. Image 3 corresponds to Fig 3 in manuscript. Western blot analysis of Dermatopontin protein. Lane 1: protein marker; Lane 2: standard Dermatopontin; Lane 3: purified recombinant dermatopontin. Image 4A corresponds to S1A Fig in manuscript. Double digestion of expression vector under optimized conditions. Lane 1:1kb DNA ladder; Lane 2: uncut pRSETA vector; Lane 3 &4: pRSETA vector digested with 0.5 μl of Bam HI and Hind III enzyme at 37°C for 0.5 h and 1 h respectively. Image 4B corresponds to S1B Fig in manuscript. Double digestion of DPT gene. Lane 1: 100 bp DNA ladder; Lane 2: uncut Dermatopontin gene; Lane 3: empty lane; Lane 4–6: Dermatopontin gene digested with 0.5 μl of Bam HI and Hind III enzyme at 37°C for 1 h. All gel images were documented using Bio-Rad gel documentation system (Universal Hood II Gel Doc System, Bio-Rad, USA). Agarose gels were observed under UV light and SDS-gels were observed under white light illumination in the Gel Doc system. The imaging of nitrocellulose membrane probed with DPT antibody was done in Odyssey Fc imager LI-COR, USA after addition of chemiluminescence reagent on to the membrane.
(PDF)
