# Supplementary figures and images for: Cloning, expression and purification of recombinant dermatopontin in Escherichia coli
Source: PLoS One. 2020 Nov 30;15(11):e0242798. doi: 10.1371/journal.pone.0242798 (PMC7703894; doi:10.1371/journal.pone.0242798)

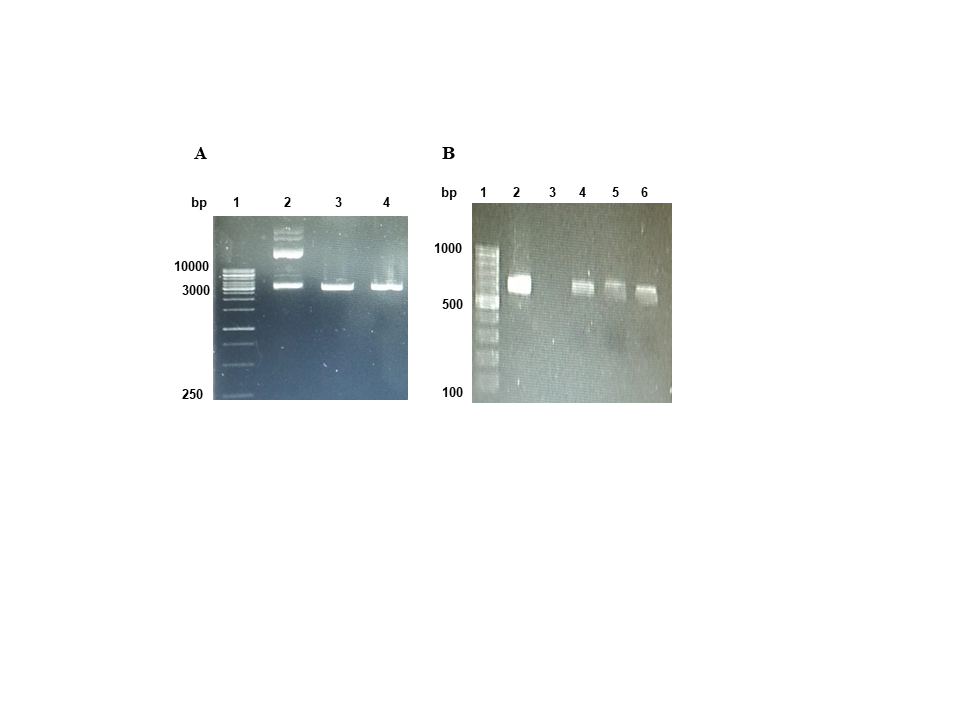

Supplement: S1 Fig — (A) Lane 1: 1kb DNA ladder; Lane 2: uncut pRSETA vector; Lane 3 &4: pRSETA vector digested with 0.5 μl of Bam HI and Hind III enzyme at 37°C for 0.5 h and 1 h respectively, (B) Lane 1: 100 bp DNA ladder; Lane 2: uncut Dermatopontin gene; Lane 3: empty lane; Lane 4–6: Dermatopontin gene digested with 0.5 μl of Bam HI and Hind III enzyme at 37°C for 1 h. (TIF) [file pone.0242798.s001.tif]

Image 1

A

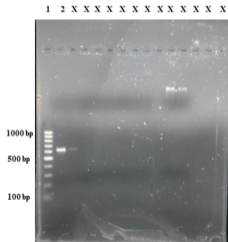

B

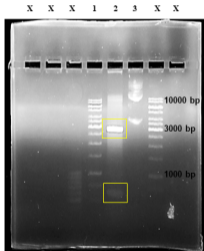

C

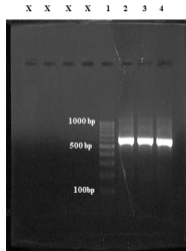

Image 2

A

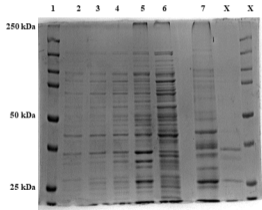

B

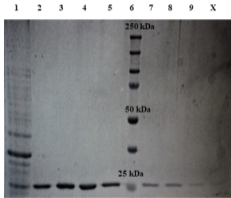

C

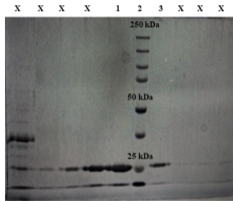

**Image 3**

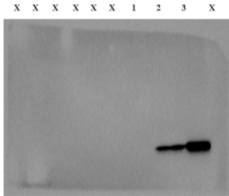

**Image 4**

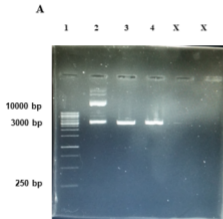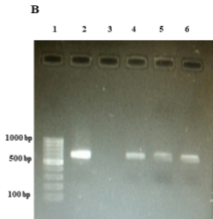

Supplement: S1 Raw images — Image 1A corresponds to Fig 1A in manuscript. PCR amplification of Dermatopontin gene. Lane 1: 100 bp DNA ladder; Lane 2: Dermatopontin gene fragment of 614 bp size amplified from the synthetic construct using gene specific primers. Image 1B corresponds to Fig 1B in manuscript. Transformation confirmation by double digestion of recombinant vector. Lane 1: 1kb DNA ladder; Lane 2: Restriction digestion of recombinant vector (pRSETA- DPT) with enzymes Bam HI and Hind III resulted in vector fragment and DPT gene fragment; Lane 3: uncut recombinant vector. Image 1C corresponds to Fig 1C in manuscript. Transformation confirmation by colony PCR. Lane 1: 100 bp DNA ladder; Lane 2–4: Dermatopontin gene amplified from transformed clone by colony PCR. Image 2A corresponds to Fig 2A in manuscript. SDS PAGE analysis of rDPT expressed in E.coli GJ1158 host as inclusion bodies. Lane 1: protein marker; Lane 2: Protein lysate of host transformed with empty vector (pRSETA); Lane 3: induced host transformed with recombinant vector; Lane 4: soluble protein fractions obtained after homogenization of induced host cells transformed with recombinant vector; Lane 5: insoluble protein fractions obtained after homogenization of induced host cells transformed with recombinant vector; Lane 6: uninduced host transformed with recombinant vector; Lane 7: urea solubilised inclusion body fraction. Image 2B corresponds to Fig 2B in manuscript. SDS PAGE analysis of rDPT purified by affinity chromatography. Lane 1: Flow through from nickel activated Sepharose column; Lane 2,3,4,5,7,8,9: Purified refolded rDPT eluted at 150 mM imidazole concentration; Lane 6: protein marker. Image 2C corresponds to Fig 2C in manuscript. SDS PAGE analysis of centricon concentrated DPT protein. Lane 1 & 3: centricon concentrated rDPT elutes; Lane 2: protein marker. Image 3 corresponds to Fig 3 in manuscript. Western blot analysis of Dermatopontin protein. Lane 1: protein marker; Lane 2: standard Dermatopontin; Lane 3: pur [file pone.0242798.s002.pdf]
